# Supplementary material for: The field and resistance training loads of academy rugby league players during a pre-season: Comparisons across playing positions
Source: PLoS One. 2022 Aug 9;17(8):e0272817. doi: 10.1371/journal.pone.0272817 (PMC9362933; doi:10.1371/journal.pone.0272817)
Supplement: Attachment — Submitted filename: Response To Reviewers.docx [file pone.0272817.s001.docx]

PONE-D-22-08332

The Field and Resistance Training Loads of Academy Rugby League Players During a Pre-Season: Comparisons Across Playing Positions

June 2022

Dear Academic Editor

Thank you for your recent review in relation to the submission of the above article for PLOS One

All reviewer comments were helpful and have allowed us to improve a number of areas in the paper.

We have addressed the majority of the comments using track changes within the paper (and include a clean copy) alongside a response to each of the reviewer’s comments below in relation to the changes that have occurred. Line numbers below refer to the changes in the track changes document. I hope this is useful in considering our revised submission.

We have also addressed the editing requirements of the journal including headings, references, etc. The study’s minimal underlying data set is now available via public repository at: https://doi.org/10.25448/lbu.20075939.v1

We hope all the below responses and changes to the paper are to the standard expected and hope that the paper is accepted to the journal in the near future.

Yours Sincerely

David Anthony Moore

On Behalf of all co-authors

Reviewer #1:

I appreciate the opportunity to comment to the authors on their manuscript titled "The Field and Resistance Training Loads of Academy Rugby League Players during a Pre-Season: Comparisons Across Playing Positions". The manuscript's issue is interesting and relevant in the current context of knowledge at the level of youth training in Rugby. I consider that the relevance of the study lies in the limited research available that explores the training load of academy rugby league players.
The purpose of the study was to quantify the field and resistance training loads of academy rugby league players during a pre-season period and compare training loads between playing positions (i.e., forwards vs. backs).The study is well structured, with the results adequately discussed.
I do however have some concerns that I point out to below:

**Thank you for your constructive comments, we have addressed each comment below, and feel it has strengthened the manuscript.**

Please indicate the power sample calculation. Power calculations allow the reader to know how many subjects are required in order to avoid a type I or a type II error.

**Due to the observational case study nature of the current research, the sample size utilised was that which was available based upon the applied nature of this study. This study adopted a convenience sample, therefore an a priori power sample calculation was not performed. In terms of our sample size justification, we acknowledge resource constraints as our rationale, in accordance with suggestions by Lakens (2022). We appreciate this comment and have added the following sentence on lines 127-128 to make this clear for the reader:**

*“Due to the applied nature of this research, the number of participants included was dictated by the resource constraints of the professional sporting organisation (28).”*

Line 137-138 - I am of the opinion that it is more appropriate to cite for the determination of the s-RPE the reference of : Foster, C, Florhaugh, JA, Franklin, J, Gottschall, L, Hrovatin, LA, Parker, S, Doleshai, P, Dodge, C. A new approach to monitoring exercise training. J Strength Cond Res 15: 109-115, 20 2001.

**We have added the above citation for determination of s-RPE now on line 147.**

Line 157 and remaining text - please correct the units of the speed threshold (m/s) and also the average speed (m/min)

**m·s^-1^ and m·min^-1^ have now been applied throughout the manuscript.**

A comment for future studies - the repetition method is a very simple method for estimating training volume but it offers a poor estimate of the amount of work or volume of training completed in a resistance training bout or training programme. A more accurate approach would be to either directly quantify work accomplished, or use estimates which account for the actual weight lifted by the athlete when attempting to either quantify or equate training loads when comparing various training interventions.

**Thank you for your comment, which we agree with. However, this study provides the first attempt at quantifying both gym and field training loads in academy rugby league. Due to the study design, we were only able to report repetition method for the resistance training loads alongside the resistance training sRPE. We have acknowledged this as a limitation of the study in the discussion (lines 342-344 & 371-377):**

*“In summary, while providing an important starting point for the study of resistance training practices in academy rugby league, reports based on repetition volume leave out relevant details that dictate the adaptive response.” “Quantifying resistance exercise remains a challenge, numerous independent variables that elicit adaptation were unable to be captured (e.g. type of exercise, relative load lifted, inter-set periods, and repetition velocity). While the current study reported on repetition volume, the addition of sRPE attempts to capture the “load” associated with gym related content. Advancements in technology and data management enable the ability to capture the different constructs that influence performance outcomes which future studies may wish to explore.”*

**Despite making up a large proportion of the total training load, the load imposed by resistance training in a team sport environment remains poorly understood. A universal method monitoring the training load of resistance training is required but remains a challenge largely due to practical constraints.**

Please clarify why the authors did not use the acceleration density index metric that allows quantifying the intensity of speed change activity exclusively during locomotive work, rather than the whole time period. In my opinion this might be preferred to acceleration density as a way to analyze speed change demands excluding rest periods.

**This is a really interesting point. The purpose of this paper was to capture overall training load, acceleration density provides an average of absolute acceleration values (quantifies the intensity of speed change activity) over time. In addition to the other GPS metrics we reported (total distance, high speed meters, average m·min^-1^, PlayerLoad, and tackles), the inclusion of acceleration density achieves our aim of capturing overall training load.**

**Additionally, previous reviews examining the use of wearable GPS devices for quantifying activity demands have identified a lack of consistency and consensus in the methodological procedures used across studies. As the reliability and usefulness of high-intensity acceleration and deceleration data can be enhanced by careful consideration to the data processing technique used, future research is still required to establish which metric and data processing methods provide the most valid, reliable and sensitive data outputs. With respect to this, an average acceleration metric, calculated by taking the absolute value of all raw values and averaging them over the duration of a selected time period, has been found to have better reliability and sensitivity across a range of GPS devices than threshold-based approaches (Delaney et al., 2018; Harper et al., 2019).**

**If the focus of the study was on the intensity of specific training drills, acceleration density index may be appropriate, however, for the purposes of this paper we would respectfully prefer to keep the acceleration density metric.**

Line 163, 332 and 357- for confirming whether it is actually the reference [31] Varley et al (2012) that the authors intend to cite, since it does not address the repetition method.

**We intended to cite Scott et al. (2016) and have made this correction (now lines 172, 338, 373).**

Please standardize throughout the text the way in which the p-value is expressed, e.g., 0.00 or .00

**Thank you for brining to our attention, we have amended to reflect a standardized p-value throughout (p < 0.001).**

In the results section, I suggest indicating the size effects of the comparisons made and with statistically significant differences.

**As suggested, we have included effect sizes alongside the statistically significant differences and believe this will strengthen the manuscript.**

I suggest that the figures could be improved in terms of their sharpness.

**As suggested, I have adjusted the resolution of the figures and achieved improved sharpness.**

In figure 4 (A) Lower Body Repetitions volume, are the data from the two positional groups presented?

**Yes, both positional groups are presented. Potentially a limitation of quantifying resistance training via the repetition method, as eluded to earlier. Due to the methodology (repetition method), study design (retrospective), and data made available (phase specific programme templates), a group mean was assumed. Repetitions were calculated weekly for upper and lower body exercises respectively. The variation in exposures between positional groups came from the number of sessions each individual player completed in each week. For the most part, both positional groups completed the same sessions across the observation window, as a result they are overlapping. It is our hope, that by improving the sharpness of the figures this is made clear.**

References

Reference [55] is not cited in the text:
Tee JC, Lambert MI, Coopoo Y. GPS comparison of training activities and game demands of professional rugby union. Int J Sport Sci Coach. 2016;11(2):200–11

**Thank you for bringing this to our attention, addressed as a reference manager issue, reference [55] has been updated and is now cited as [56] on line 406.**

Reviewer #2:

The paper is well written and provides a clear and accurate representation of the training demands of adolescent rugby players. I recommend this paper for publication.

**Thank you for your encouraging comments.**
